# Coagulation Profile in COVID-19 Patients and its Relation to Disease Severity and Overall Survival: A Single-Center Study

**DOI:** 10.3389/bjbs.2022.10098

**Published:** 2022-04-08

**Authors:** Amal Ezzat Abd El-Lateef, Saad Alghamdi, Gamal Ebid, Khalid Khalil, Saeed Kabrah, Muhammad Tarek Abdel Ghafar

**Affiliations:** ^1^ Department of Clinical Pathology, Faculty of Medicine, Tanta University, Tanta, Egypt; ^2^ Department of Laboratory Medicine, Faculty of Applied Medical Science, Umm Al-Qura University, Makkah, Saudi Arabia; ^3^ Department of Laboratory Medicine, Security Forces Hospital, Makkah, Saudi Arabia; ^4^ Department of Clinical Pathology, National Cancer Institute, Cairo University, Cairo, Egypt; ^5^ Department of Internal Medicine, Security Forces Hospital, Makkah, Saudi Arabia

**Keywords:** Covid-19, coagulopathy, D-dimer, factor VIII, von Willebrand factor, ristocetin cofactor

## Abstract

**Objectives:** This study aims to investigate hemostatic changes in patients with coronavirus disease (COVID-19) and their relationship to disease severity and survival.

**Methods:** This study included 284 patients with COVID-19 who attended the Security Forces Hospital, Makkah, Saudi Arabia between October 2020 and March 2021, and retrospectively reviewed their demographic, radiological, and laboratory findings. The coagulation profile was assayed at the time of diagnosis for platelet counts using an automated hematology analyzer; Sysmex XN2000 while international normalized ratio (INR), activated partial thromboplastin time (aPTT), fibrinogen, D-dimer, factor VIII, ristocetin cofactor (RiCoF), and von Willebrand factor antigen (VWF-Ag) were measured by Stago kits on a Stago automated coagulation analyzer (STA Compact Max^®^).

**Results:** In this study, 32.3% of the cases had severe disease, while 8.8% of the cases died. D-dimer, factor VIII, and RiCoF were the only independent predictors of disease severity, with factor VIII and RiCoF having significantly higher areas under the curve (AUCs) than D-dimer (all *p* < 0.001). Furthermore, age, aPTT, and factor VIII were associated with an increased risk of mortality in multivariate Cox regression analysis, with factor VIII having a higher AUC of 0.98 than aPTT with an optimal cut-off value of >314 IU/dl in predicting mortality. Cases with factor VIII levels >314 IU/dl, compared to those with factor VIII levels <314 IU/dl, were associated with a significantly shorter mean overall survival time (20.08 vs. 31.35 days, *p* < 0.001), a lower survival rate (30.3% vs. 99.2%, *p* < 0.001), and a 16.62-fold increased mortality risk.

**Conclusion:** RiCoF is a novel predictor of disease severity in COVID-19, while factor VIII is confirmed as a predictor of severity and mortality in COVID-19 patients and is associated with lower overall survival and increased mortality risk.

## Introduction

The coronavirus disease (COVID-19) appeared in Wuhan, Hubei Province, China in 2019, and is caused by the severe acute respiratory syndrome coronavirus 2 (SARS-CoV-2) (1). SARS-CoV-2 is a beta genus belonging to the Coronavirinae subfamily of Coronaviridae (2). The infection usually spreads through respiratory secretions during coughing, sneezing, or contact with contaminated surfaces. Clinical presentations range from moderate upper respiratory symptoms to severe or critical manifestations, including respiratory distress, coagulopathy, and multiple organ dysfunction leading to death (3). The severity of COVID-19 is linked to many risk factors, including immunodeficiency, old age, pregnancy, and comorbidities such as diabetes, malignancy, and chronic lung or kidney disease (4). Therefore, early determination of severity is very important to guide the treatment of COVID-19, prevent the development of its complications, and reduce mortality.

Coagulopathy is one of the most common hematological complications of COVID-19, which is mainly associated with venous thromboembolism, multiple organ failure, and poor prognosis (5). Venous thromboembolism was detected in 40% of hospitalized patients with COVID-19 and 22.5% of those treated with prophylactic anticoagulants (6). Although the exact pathogenesis of coagulopathy in COVID-19 has not been fully elucidated, some factors could contribute to the hemostatic changes in COVID-19, including cytokine storm, neutrophil activation, impaired endothelial function, platelet activation, tissue factor expression, and coagulation induction (7).

In COVID-19, coagulopathy is manifested by changes in coagulation factors similar to those of sepsis-induced coagulation and can progress to an atypical form of disseminated intravascular coagulation (DIC) lacking thrombocytopenia or hypofibrinogenemia (8). Abnormalities of hemostatic factors such as D-dimer at the time of admission were discovered to be associated with disease severity and mortality in Chinese patients with COVID-19 (7). Coagulation profile testing can be used to predict prognosis, plan treatment for COVID-19, identify patients who will need anticoagulants, those who will need antiplatelets, and adjust the dose and duration of medications (9). In addition, routine coagulation tests are inexpensive and can be used to detect disease severity in COVID-19 patients in areas with limited resources (10).

COVID-19 is characterized by an abnormal DIC that differs from the typical DIC and shows abnormalities in coagulation markers that are inconsistent between studies with respect to their levels in COVID-19 patients as well as their relationship to disease severity and related mortality (7, 11). Furthermore, there is limited data about other important coagulation factors that may be linked to coagulopathy in COVID-19, like the ristocetin cofactor (RiCoF). This makes it difficult to control the disease and reduce its severity and mortality.

Endothelial cell activation, the major player in COVID-19 associated coagulopathy and venous thrombosis, is mediated either directly *via* SARS-CoV-2 infection of the vascular endothelium or indirectly by cytokine storm, complement activation, or hypoxia-induced by SARS-CoV-2, with subsequent release of factor VIII and von Willebrand factor (VWF) (12). So, we speculated that the coagulation factors, especially factor VIII and VWF, can be increased in COVID-19 patients and can be related to thromboembolic manifestations exacerbating the condition in these patients. Therefore, we conducted this study to investigate the hemostatic changes in patients with COVID-19 with a particular focus on coagulation factors such as factor VIII, von Willebrand factor antigen (VWF-Ag), and RiCoF and their relationships to disease severity and patient survival.

### Subjects and Methods

This retrospective study included a total of 284 confirmed patients with COVID-19 who attended the Security Forces Hospital, Makkah Al Mukarramah, Saudi Arabia between October 2020 and March 2021. Since the thromboembolic frequency is 5.6% (13) and the expected number of patients with COVID-19 attending the Security Forces Hospital is 1200, the adequate sample size was calculated to be 214. The sample was calculated using Open Epi software with a confidence limit of 2.8 and a design effect of 1. All cases were newly diagnosed by nasopharyngeal swabs with reverse transcriptase real-time polymerase chain reaction (RT-PCR) for SARS-CoV-2 according to the guidelines of the World Health Organization (WHO) (14). Patients with viral infections other than SARS-CoV-2, incomplete radiological and/or laboratory data, hereditary or acquired coagulopathy, pregnancy, those who received anticoagulants prior to blood sampling, or those who have comorbidities such as cardiovascular disorders, diabetes mellitus, chronic lung diseases, kidney diseases, liver diseases, cancer, and cerebrovascular diseases were excluded from this study.

Patients were treated according to their disease severity, as recommended by the Guidelines for Supportive Care and Antiviral Therapy for COVID-19 by the Ministry of Health in the Kingdom of Saudi Arabia (15). Non-severe cases were treated symptomatically at home, whereas severe and critical cases were admitted to the hospital and treated according to their clinical condition. Severe cases received treatment, including antiviral therapy (Lopinavir/Ritonavir, Ribavirin, or Favipiravir), interferon beta-1b, and corticosteroids. Critical cases were admitted to the intensive care unit (ICU) and received antiviral therapy (Remdesivir or Favipiravir), corticosteroids, and tocilizumab for cases of cytokine release syndrome. When necessary, thromboprophylaxis with antibiotics and antifungals is considered in addition to other supportive care measures, including active oxygen therapies such as nasal catheterization, oxygen masks, and high-flow oxygen therapy, as well as respiratory and circulatory support.

The outcomes of this study were disease severity and overall survival. COVID-19 cases were divided according to WHO guidelines (14) based on their disease severity into four grades that include mild, moderate, severe, and critical disease. The disease is considered severe if clinical symptoms of pneumonia (fever, cough, shortness of breath, and tachypnea) are present with any of the following: respiratory rate >30 breaths/min, oxygen saturation <90% in room air, or signs of acute respiratory distress. It is considered a serious disease once acute respiratory distress syndrome, sepsis, or multiorgan failure develops. In this study, COVID-19 patients with mild and moderate diseases were included in one group, the “non-severe group,” while severe and critical cases were included in another group, the “severe/critical group.” The overall survival of COVID-19 patients who did not die as survivors or died as non-survivors was determined.

The demographic, radiological, and routine laboratory data at the time of diagnosis before receiving any treatment were obtained and collected from the hospital’s electronic patient registry. Demographic data included age and gender, while radiological data included the location and pattern of lung infiltrations as determined by chest computed tomography (CT). Routine laboratory data, according to our local COVID-19 protocol, included lactate dehydrogenase (LDH), blood cell counts with absolute neutrophil and lymphocyte counts, and acute phase reactants such as C-reactive protein and ferritin. The coagulation profile was assessed at the time of diagnosis before any treatment was received and included platelet counts, international normalized ratio (INR), activated partial thromboplastin time (aPTT), fibrinogen, D-dimer, and coagulation factors such as factor VIII, RiCoF, and VWF-Ag.

From all patients, 10 ml of venous blood were collected and delivered into EDTA and 3.2% citrated vacutainer tubes. All samples were collected upon the patient’s diagnosis. All study participants did not receive any anticoagulant medication before blood samples were drawn. Platelet counts were determined using an automated hematology analyzer, the Sysmex XN2000 (Japan). A coagulation assay including INR, aPTT, fibrinogen, D-dimer, factor VIII, RiCoF, and VWF-Ag was performed using Stago kits (STA®-NeoPTima, Catalog no: 01165, STA®-PTT 5, Catalog no: 00595, STA®-Liquid Fib, Catalog no: 00673, STA®-Liatest^®^ D-Di PLUS, Catalog no: 00662, STA®-ImmunoDef VIII, Catalog no: 00728, STA^®^-VWF: RCo, Catalog no: 01191, STA®-Liatest^®^ VWF: Ag, Catalog no: 00518, respectively) on a Stago automated coagulation analyzer (STA Compact Max^®^, France) according to the manufacturer’s instructions. The RiCoF assay, in particular, is based on the change in turbidity of a platelet suspension, which is measured photometrically. The intra-assay and inter-assay coefficients of variation of RiCoF are 7.0% and 13.3%, respectively. The laboratory analyses were performed in accordance with the manufacturer’s recommendations and laboratory standard operating procedures.

The study was conducted in accordance with the Declaration of Helsinki II. The protocol was approved by the Ethics Committee of the Security Forces Hospital in Makkah, KSA (IRB No. 0394-040121).

### Statistical Analysis

The data were analyzed using the Statistical Package for the Social Sciences (SPSS) version 20.0 (Armonk, NY, United States: IBM Corp) and MedCalc Statistical Software version 15.8 (MedCalc Software bvba, Ostend, Belgium). Numerical data were presented as mean and standard deviation, or median and interquartile range. They were tested for normality using the Kolmogorov-Smirnov test and compared between severe and non-severe groups, as well as survivors and non-survivors, using the Student’s t-test for normally distributed variables and the Mann-Whitney U test for non-normally distributed variables. Categorical data were presented as numbers and compared between groups using the Chi-square χ^2^ test. The severity and overall survival were considered dependent variables, while demographic, radiological, and laboratory data were considered independent variables in this study. Predictors of disease severity were evaluated by binary logistic regression analysis, and then significant indicators were analyzed by multivariate logistic regression. Univariate and multivariate Cox regression analyses were used to assess prognostic factors for overall survival. The Kaplen-Meier analysis was used to calculate the overall survival rate and time, and the log-rank test was used to determine significance. The predictive efficacy was determined using receiver operating characteristic (ROC) curve analysis and the areas under the curve (AUCs) for coagulation markers were calculated and compared according to DeLong et al. (16). The optimal cut-off values were determined by Youden’s index. All p-values were two-tailed and were considered significant if they were less than 0.05.

## Results

In this study, a total of 407 patients newly diagnosed with COVID-19 were retrospectively reviewed, and 123 patients were excluded due to incomplete laboratory data, the presence of comorbidities, pregnancy, or receiving medication prior to sampling. Finally, 284 patients with COVID-19 were included. There were 32.3% of patients with severe disease (92 of 284 cases), and 8.8% of cases died (25 of 284). The demographic, radiological, biochemical, inflammatory, and coagulation findings of the COVID-19 patients included in this study are presented in Table 1.

**TABLE 1:** Basic demographic, radiological, and laboratory characteristics of the studied COVID-19 patients.

	References range (Upper-lower limits)	Total *n* = 284	Non-severe *n* = 192	Severe *n* = 92	P-value	Survivor *n* = 259	Non-survivor *n* = 25	P-value
Age (Years)	**—**	52.7 ± 18.2	50.7 ± 18.6	56.9 ± 16.7	0.007*	51.4 ± 18.0	66.1 ± 14.7	<0.001*
Sex (Male/Female)	**—**	162/122	100/92	62/30	0.015*	147/112	15/10	0.755
Radiological Findings								
Infiltrations (Yes/No)	**—**	154/130	68/124	86/6	<0.001*	130/129	24/1	<0.001*
Site (Unilateral/Bilateral)	**—**	61/93	50/18	11/75	<0.001*	60/70	1/23	<0.001*
Pattern (Focal/Diffuse)	**—**	47/107	39/29	8/78	<0.001*	45/85	2/22	0.010*
LDH (U/l)	230.0–460.0	274.5 (177.8)	229.0 (102.5)	429.5 (211.8)	0.008*	265.0 (151.0)	528.0 (325.0)	<0.001*
Ferritin (ng/ml)	30.0–400.0	422.6 (843.5)	276.1 (469.9)	1088.5 (1199.5)	0.001*	253.3 (706.2)	1370.0 (1267.8)	<0.001*
Inflammatory Profile								
CRP (mg/l)	0–5.0	2.8 (8.4)	1.6 (3.3)	11.4 (11.9)	<0.001*	2.5 (6.5)	10.9 (11.2)	<0.001*
Total WBCs (x 10^9^/l)	4.0–11.0	7.07 (4.61)	6.51 (4.07)	8.59 (8.21)	<0.001*	6.75 (4.36)	16.95 (18.97)	<0.001*
Absolute neutrophil counts (x10^9^/l)	2.50–6.00	4.69 (4.43)	4.21 (3.77)	6.24 (7.66)	<0.001*	4.51 (3.98)	15.64 (17.96)	<0.001*
Absolute lymphocytic counts (x10^9^/l)	1.00–4.80	1.37 (0.92)	1.49 (0.96)	1.17 (0.74)	0.003*	1.40 (0.97)	1.03 (0.76)	<0.001*
Coagulation profile								
Platelets counts (x 10^9^/l)	150–450	227 (112)	236 (101)	219 (142)	0.176	236 (110)	117 (160)	<0.001*
INR	0.80–1.10	1.11 (0.14)	0.99 (0.10)	1.12 (0.28)	<0.001*	1.00 (0.12)	1.36 (0.66)	<0.001*
aPTT (Sec)	26.0–40.0	35.7 (11.0)	33.0 (6.0)	46.0 (20.0)	<0.001*	34.7 (9.0)	49.0 (36.8)	<0.001*
D-dimer (µg/ml)	0–0.50	0.80 (1.90)	0.60 (0.60)	3.00 (4.93)	<0.001*	0.70 (1.30)	6.10 (12.55)	<0.001*
Fibrinogen (mg/dl)	200.0–400.0	259.0 (205.0)	210.0 (90.0)	500.0 (210.0)	<0.001*	243.3 (165.0)	603.0 (240.0)	<0.001*
Factor VIII (IU/dl)	50.0–150.0	120.0 (156.5)	110.0 (25.0)	290.0 (63.8)	<0.001*	120.0 (90.0)	340.0 (29.0)	<0.001*
RiCoF (IU/dl)	50.0–200.0	125.0 (176.5)	120.0 (15.0)	310.0 (79.5)	<0.001*	120.0 (60.0)	354.0 (62.5)	<0.001*
VWF-Ag (IU/dl)	50.0–150.0	215.0 (150.0)	200.0 (40.0)	415.5 (221.8)	<0.001*	210.0 (113.0)	510.0 (149.5)	<0.001*

n, number; CRP, C-reactive protein; LDH, lactate dehydrogenase; WBCs, white blood cells; INR, international normalized ratio; aPTT, activated partial thromboplastin time; RiCoF, ristocetin cofactor; VWF-Ag, von Willebrand Factor Antigen.

Data are presented as Mean ± Standard Deviation or median (interquartile range) or number.

* Statistically significant at *p* ≤ 0.05.

In terms of disease severity, there were significant differences in the levels of biochemical, inflammatory, and coagulation markers between non-severe and severe/critical cases, with significant increases in LDH, ferritin, CRP, total WBCs, absolute neutrophil counts, INR, aPTT, D-dimer, fibrinogen, factor VIII, RiCoF, and VWF-Ag, and a significant decrease in the absolute lymphocyte counts in severe/critical cases compared with those with non-severe disease, with no significant difference in the platelet counts. Moreover, cases with severe/critical disease showed a higher degree of diffuse infiltration involving the lung lobes (Table 1).

**FIGURE 1:**
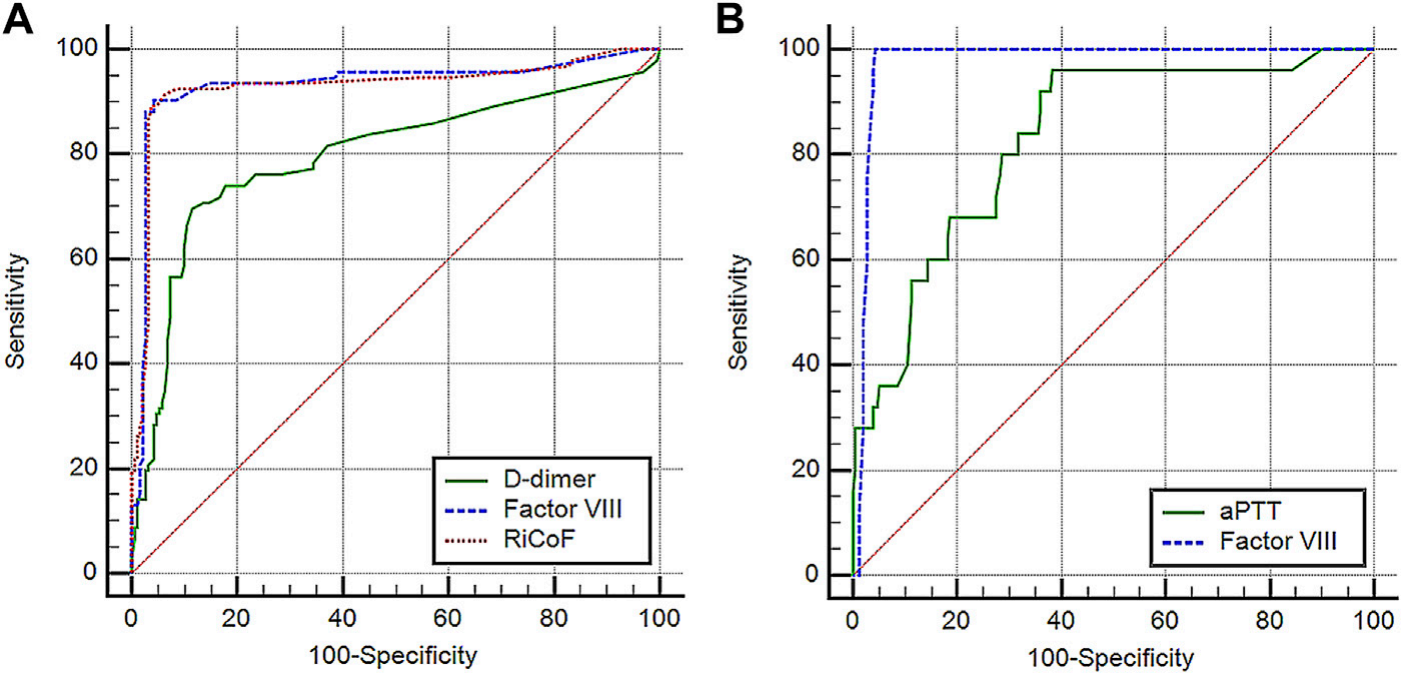
Performance characteristics of coagulation markers in COVID-19 patients: **(A)** ROC curve analysis of D-dimer, factor VIII, and RiCoF for predicting COVID-19 disease severity, **(B)** ROC curve analysis of aPTT and factor VIII for predicting COVID-19 mortality.

All previous inflammatory and coagulation markers, as well as CT characteristics, were significantly related to disease severity in univariate analyses. However, multivariate logistic regression analysis revealed that D-dimer, factor VIII, and RiCoF were the only independent predictors of disease severity (Table 2). The predictive value of these coagulation markers was determined using ROC curve analysis (Figure 1A). The AUC of D-dimer is 0.79 (95% CI: 0.74–0.84) with a sensitivity of 69.6%, a specificity of 88.5%, and an optimal cut-off value of >1.7 μg/ml. Factor VIII has an AUC of 0.93 (95% CI: 0.90–0.96) with a sensitivity of 90.2%, a specificity of 95.8%, and an optimal cut-off value of >180 IU/dl, while RiCoF has an AUC of 0.93 (95% CI: 0.89–0.97) with a sensitivity of 89.1%, a specificity of 96.4%, and an optimal cut-off value of >170 IU/dl. Factor VIII and RiCoF have significantly higher AUCs than D-dimer (all *p* < 0.001).

**TABLE 2:** Multiple regression analysis of predictors of disease severity and mortality in COVID-19 patients.

	Disease severity				Disease mortality			
	Univariate analysis		Multivariate analysis		Univariate analysis		Multivariate analysis	
	OR (95% CI)	P-value	OR (95% CI)	P-value	HR (95% CI)	P-value	HR (95% CI)	P-value
Age (Years)	1.21 (1.05–1.40)	0.008*	0.94 (0.56–1.60)	0.944	1.44 (1.09–1.91)	0.011*	2.05 (1.25–3.35)	0.004*
Sex (Male/Female)	1.90 (1.13–3.2)	0.015*	1.68 (0.28–10.21)	0.576	1.21 (0.53–2.76)	0.649	**—**	**—**
Radiological Findings								
Infiltrations (Yes/No)	26.14 (10.85–62.94)	<0.001*	5.41 (0.99–38.55)	0.102	7.94 (1.02–61.75)	0.048*	5.94 (0.88–55.76)	0.112
Site (Bilateral vs. Unilateral)	18.94 (8.25–43.48)	<0.001*	1.67 (0.19–15.00)	0.469	2.37 (0.32–17.63)	0.401	**—**	**—**
Pattern (Diffuse vs. Focal)	12.78 (5.33–30.60)	<0.001*	4.86 (0.71–33.11)	0.106	1.57 (0.37–6.73)	0.547	**—**	**—**
Severity (Severe/Critical vs. Non-severe)	**—**	**—**	**—**	**—**	3.11 (1.02–9.49)	0.046*	1.74 (0.28–10.91)	0.557
LDH (U/l)	1.37 (1.09–1.72)	0.007*	0.75 (0.40–1.40)	0.362	1.16 (1.04–1.32)	0.014*	1.02 (0.96–1.09)	0.524
Ferritin (ng/ml)	1.31 (1.19–1.44)	<0.001*	1.06 (0.94–1.13)	0.571	1.02 (0.99–1.05)	0.162	**—**	**—**
Inflammatory Profile								
CRP (mg/l)	1.41 (1.28–1.55)	<0.001*	0.98 (0.87–1.28)	0.906	0.96 (0.88–1.06)	0.418	**—**	**—**
Total WBCs (x 10^9^/l)	1.13 (1.07–1.19)	<0.001*	0.91 (0.66–1.11)	0.318	1.05 (1.01–1.09)	0.027*	1.61 (0.98–3.64)	0.009*
Absolute neutrophil counts (x 10^9^/l)	1.12 (1.07–1.18)	<0.001*	0.42 (0.09–2.00)	0.273	1.05 (1.01–1.10)	0.014*	3.61 (1.31–9.97)	0.011*
Absolute lymphocytic counts (x 10^9^/l)	0.65 (0.46–0.92)	0.016*	0.37 (0.40–3.11)	0.359	0.53 (0.25–1.13)	0.102	**—**	**—**
Coagulation profile								
Platelets counts (x 10^9^/l)	0.92 (0.73–1.18)	0.517	**—**	**—**	0.62 (0.39–0.98)	0.039*	0.76 (0.45–1.30)	0.318
INR	1.47 (1.25–1.74)	<0.001*	1.05 (0.82–1.35)	0.692	1.07 (1.02–1.12)	0.011*	0.94 (0.83–1.07)	0.355
aPTT (sec)	1.41 (1.29–1.55)	<0.001*	1.12 (0.96–1.19)	0.298	1.08 (1.05–1.12)	<0.001*	1.10 (1.05–1.15)	<0.001*
D-dimer (µg/ml)	1.27 (1.16–1.40)	<0.001*	0.75 (0.58–0.97)	0.028*	1.04 (0.98–1.10)	0.190	**—**	**—**
Fibrinogen (mg/dl)	1.84 (1.59–2.13)	<0.001*	1.37 (0.88–2.13)	0.167	1.04 (0.96–1.12)	0.372	**—**	**—**
Factor VIII (IU/dl)	1.83 (1.62–2.08)	<0.001*	1.58 (1.11–2.26)	0.011*	1.42 (1.15–1.74)	0.001*	1.32 (1.08–2.14)	0.016*
RiCoF (IU/dl)	1.67 (1.50–1.86)	<0.001*	1.63 (0.98–2.72)	0.040*	1.12 (1.03–1.21)	0.006*	0.96 (0.69–1.32)	0.788
VWF-Ag (IU/dl)	1.40 (1.29–1.52)	<0.001*	0.82 (0.58–1.15)	0.245	1.11 (1.04–1.18)	0.001*	1.05 (0.86–1.29)	0.629

OR, odds ratio; HR, hazards ratio; CI, confidence interval; CRP, C-reactive protein; LDH, lactate Dehydrogenase; WBCs, white blood cells; INR, international normalized ratio; aPTT, activated partial thromboplastin time; RiCoF, ristocetin cofactor; VWF-Ag, von Willebrand Factor Antigen.

All variables with *p* < 0.05 was included in the multivariate.

* Statistically significant at *p* ≤ 0.05.

There were significant differences in CT findings as well as levels of biochemical, inflammatory, and coagulation markers between survivors and non-survivors. Compared to the survivors, non-survivors showed a significant increase in LDH, ferritin, CRP, total WBCs, absolute neutrophil counts, INR, aPTT, D-dimer, fibrinogen, factor VIII, RiCoF, and VWF-Ag and a significant decrease in platelet counts and absolute lymphocyte counts (Table 1). Furthermore, non-survivors showed a high frequency of bilateral diffuse lung infiltrations.

In a univariate Cox regression analysis, increased age, presence of lung infiltrates, higher severity score, increased LDH, total WBCs, absolute neutrophil counts, INR, aPTT, factor VIII, RiCoF, VWF-Ag, and decreased platelet counts were all associated with increased mortality risk, whereas only age, total WBCs, absolute neutrophil counts, aPTT, and factor VIII were associated with increased mortality in a multivariate Cox regression analysis (Table 2). Therefore, we tested the predictive value of these coagulation markers for mortality using ROC curve analysis (Figure 1B) and found that factor VIII has a significantly higher AUC of 0.98 (95% CI: 0.95–0.99) than aPTT (0.83 [95% CI: 0.78–0.87], *p* = 0.001).

**FIGURE 2:**
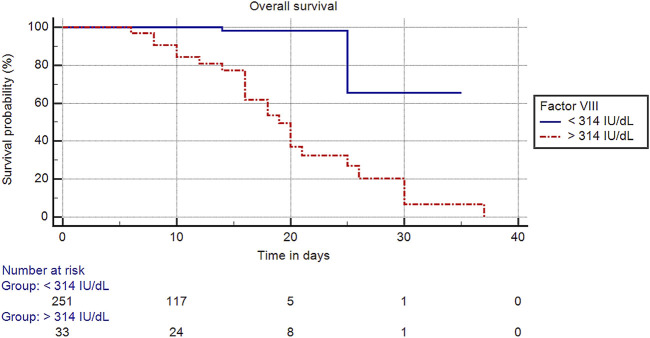
Kaplan-Meier curve of overall survival for COVID-19 according to factor VIII level.

The optimal cut-off value of factor VIII was >314 IU/dl in predicting mortality, and cases with factor VIII levels >314 IU/dl compared to those with factor VIII levels ≤314 IU/dl were associated with a significantly shorter mean overall survival time (20.08 [95% CI: 16.93–23.26] vs. 31.35 [95% CI: 26.08–36.63] days, *p* < 0.001), a lower survival rate (30.3% vs. 99.2%, *p* < 0.001) (Figure 2), and an increased risk of mortality (HR: 16.62 [95% CI: 3.68–75.04], *p* < 0.001).

## Discussion

This study was conducted to investigate coagulation markers in a large cohort of COVID-19 patients and their relationship to disease severity and mortality in order to confirm the findings of previous studies. As far as we know, this is the first study to investigate the role of RiCoF in predicting COVID-19 severity and mortality.

Thrombocytopenia occurs most commonly in DIC and is associated with organ dysfunction (17). Our study revealed that the average platelet count is normal in COVID-19 patients as reported in some previous studies (7,18). This can be explained by the presence of inflammation in the lung that leads to the secretion of thrombopoietin, which stimulates platelet production in COVID-19 patients (19). The low platelet count was detected in only 12–36% of COVID-19 patients (5, 20). Furthermore, in this study, platelet counts did not differ between severe and non-severe cases, which is inconsistent between studies. Although some studies have found that platelet counts and thrombocytopenia do not correlate with COVID-19 disease severity (11, 21), others have found a significant association between severity and platelet counts as low as 31 × 10^9^/L in a meta-analysis of 1,779 patients with COVID-19 (22), particularly when D-dimer levels reach six times normal (7). On the other hand, platelet counts were lower in non-survivors than in survivors in our COVID-19 patients, which is consistent with previous studies in this regard. These studies found thrombocytopenia is associated with a more than fivefold increased risk of death (22–24). This indicates that the typical DIC can develop in COVID-19 patients, specifically in the late stages (7). Also, secondary infection may be a cause of thrombocytopenia in critical diseases, especially in cases of mechanical ventilation (23, 24). Moreover, drugs such as heparin should not be ignored as a cause of thrombocytopenia, but they are not applicable to this study because blood samples were taken for analysis before any treatment (25).

PT, INR, and aPTT are often elevated in DIC, especially in its acute form (17). In contrast to conventional sepsis, normal PT and aPTT are usually observed in COVID-19 patients, with only 6% of them having prolonged PT and/or aPTT (3). In this study, the mean aPTT is normal in cases of COVID-19 while the INR is slightly elevated, as reported by Yin et al. (26) who found an increase in PT level in COVID-19 patients compared to controls, while no increase in Caucasian patients was detected by Fogarty et al. (11). Furthermore, aPTT and INR are more elevated in severe versus non-severe and survivors versus non-survivors in this study. Studies have shown significantly higher INR and aPTT prolongation among severely affected and critically ill COVID-19 patients (27) as well as non-survivors (28), whereas other studies did not find any significant association with disease severity or mortality (7, 18). Activation of pathogen-associated molecular pathways is induced by SARS-CoV-2 infection, which causes activation of intrinsic and extrinsic coagulation cascades, leading to increased aPTT and INR, respectively (29).

Fibrinogen, a highly specific marker for the diagnosis of DIC, can be low in severe and late DIC cases (17). The presence of hyperfibrinogenemia was detected in most of the COVID-19 patients, with a mean of 4.55 g/L (7). In this study, fibrinogen was elevated in severe COVID-19 cases as well as in non-survivors. Similar results were observed by Tang et al. (7), Fogarty et al. (11), and Long H et al. (28) regarding the severity of the disease. In contrast to our findings, the degree of elevation is more closely related to the level of interleukin (IL)-6 than to the mortality rate (7,30). However, progressive hypofibrinogenemia is strongly associated with mortality, and a fibrinogen level of less than 1 g/L was detected in about 29% of cases in the late stages (7). As fibrinogen is an acute phase reactant protein, its increased level in our study indicates inflammation rather than consumption, which is a typical feature of DIC. These findings support the fact that a typical DIC is not a common feature of COVID-19.

D-dimer, a highly sensitive marker of DIC (17), is elevated in 36% of COVID-19 patients with an average of 0.9 mg/L (20). In our study, D-dimer was increased in our cases upon admission. Furthermore, D-dimer is independently associated with severity risk with a predictive value of 0.79. This result was similar to studies by Tang et al. (7), Han et al. (18), and Long et al. (28) who reported a more than threefold increase in D-dimer levels above normal. In this study, D-dimer is increased in severe and non-surviving cases. Consistent with our results, increased levels of D-dimer in severe disease with a mean difference of 2.97 mg/L were detected in the pooled analysis of 4 studies with 553 patients with COVID-19 by Lippi et al. (31) as well as a mean difference of 0.60 in a recent meta-analysis by Chaudhary et al. (32). In early 2020, Escher et al. described a progressive increase in D-dimer levels in a consecutive series of five COVID-19 cases with severe disease who had been admitted to ICU. They found that D-dimer levels dropped steadily or even normalized after low molecular weight heparin was given to these cases (33–35). In previous studies, non-survivors showed an increase in D-dimer levels of more than 3.5-fold, with more than 80% of them having D-dimer levels >1 mg/L (7,23). In the early stages of COVID-19 disease, endothelial damage caused by the virus leads to hypercoagulation with subsequent release of thrombin in the absence of fibrinolysis, thus increasing the level of D-dimer (36). It can also rise because of pulmonary venous thrombosis or hypoxia-induced thrombosis caused by hyperviscosity (37).

In this study, the median VWF-Ag level was significantly elevated in COVID-19 cases while the median factor VIII and RiCoF levels fell within the normal range. Consistent with our results, previous studies showed that COVID-19 patients showed elevated levels of VWF-Ag (8, 13, 38) with no significant difference in factor VIII levels compared to non-COVID-19 patients (39). However, studies reported some different findings from our study in this regard, with elevated factor VIIIc levels (8, 13, 38, 39) and RiCoF activity being detected in COVID-19 patients (8). Furthermore, factor VIII, RiCoF, and VWF-Ag were significantly elevated in severe and non-survivors in this study. Factor VIII and RiCoF are the most important predictors of disease severity, with higher predictive values than D-dimer. Factor VIII is an independent predictor of COVID-19 mortality with a higher predictive value than aPTT and is associated with a significantly lower overall survival rate of 30.3% and a 16.62-fold increased risk of death. In this context, several studies have reported that factor VIII levels are increased among COVID-19 patients admitted to the ICU (40), with VWF-Ag being an important predictor associated with in-hospital mortality in COVID-19 patients (38). Escher et al. reported that factor VIII, as well as VWF activity and antigen, were massively elevated in a consecutive series of five COVID-19 cases with severe diseases admitted to ICU (33–35), whereas ADAMTS13 activity was normal in three cases, ruling out the possibility of thrombotic thrombocytopenic purpura in these cases. Although these cases improved following the administration of low molecular weight heparin, VWF and factor VIII levels remained elevated or mildly decreased (33). Endothelial stimulation and dysfunction are mediated by SARS-CoV-2 stimulation of the angiotensin-converting enzyme 2 receptor on the surface of endothelial cells and cytokine storm in COVID-19, resulting in the release of VWF and factor VIII from the endothelium. The active endothelium will express adhesion molecules, resulting in platelet activation, recruitment, and the formation of the platelet plug that provides an adhesion site for coagulation proteins. Tissue factor expression from damaged endothelium with platelet activation and increased levels of VWF and FVIII will create a hypercoagulable state in COVID patients with thrombin generation and fibrin clot formation (12). Furthermore, VWF and FVIII have been long recognized as acute phase reactants with elevated levels indicating endothelial dysfunction and inflammation in a variety of disorders, including coronary artery disease, autoimmune disease, trauma, and infections (41, 42). Cytokines such as tumor necrosis factor-alpha, IL-6, and IL-8 promote the release of VWF multimers and FVIII (43). Thus, the elevated VWF and FVIII levels in COVID-19 indicate the state and degree of inflammation.

This study has a number of limitations. First, it is a single-center study; however, the sample size is adequate. Second, other coagulation factors were not investigated as they were not included in the routine laboratory analysis of our patients. Third, dynamic changes in coagulation markers during the course of the disease were not investigated in this study. Fourth, it is not possible to assess the effect of treatment as a prognostic factor for survival due to the wide variety of protocols used between cases and which are applied according to their clinical condition. Finally, the VWF data have not been controlled for the ABO blood group. Therefore, other coagulation factors and dynamic changes in coagulation markers during the course of COVID-19 need to be further studied in future studies. This work represents an advance in biomedical science because it confirms the previous findings of factor VIII, D-dimer, and aPTT as predictors of severity and/or mortality of COVID-19, and presents RiCoF as a novel predictor of severity in COVID-19 patients.

## Summary Table

### What is Known About This Topic

• Coagulopathy is a common hematological feature of COVID-19 associated with venous thromboembolism, multi-organ failure, and poor prognosis.
• Coagulopathy in COVID-19 is presented by atypical disseminated intravascular coagulation with variable alterations of coagulation markers.
• Coagulation profile assay can predict the prognosis and direct treatment of COVID-19 in relation to thromboprophylaxis and anti-coagulants.

### What This Work Adds

• RiCoF is a novel predictor of disease severity in COVID-19 with higher accuracy than D-dimer.
• Factor VIII, D-dimer, and aPTT are confirmed as predictors of disease severity and/or mortality in COVID-19.
• Factor VIII >314 IU/dl is associated with lower overall survival and 16.62-fold increased mortality risk in COVID-19 patients.

## Data Availability

The original contributions presented in the study are included in the article/Supplementary Material, further inquiries can be directed to the corresponding author.

## References

[1] Zhu N Zhang D Wang W Li X Yang B Song J A Novel Coronavirus from Patients with Pneumonia in China, 2019. N Engl J Med (2020) 382(8):727–33. 10.1056/nejmoa2001017 31978945 PMC7092803

[2] Yin Y Wunderink RG. MERS, SARS and Other Coronaviruses as Causes of Pneumonia. Respirology (2018) 23(2):130–7. 10.1111/resp.13196 29052924 PMC7169239

[3] Han W Quan B Guo Y Zhang J Lu Y Feng G The Course of Clinical Diagnosis and Treatment of a Case Infected with Coronavirus Disease 2019. J Med Virol (2020) 92(5):461–3. 10.1002/jmv.25711 32073161 PMC7167012

[4] Yue H Bai X Bai X Wang J Yu Q Liu W Clinical Characteristics of Coronavirus Disease 2019 in Gansu Province, China. Ann Palliat Med (2020) 9(4):1404–12. 10.21037/apm-20-887 32692208

[5] Al-Samkari H Karp Leaf RS Dzik WH Carlson JCT Fogerty AE Waheed A COVID-19 and Coagulation: Bleeding and Thrombotic Manifestations of SARS-CoV-2 Infection. Blood (2020) 136(4):489–500. 10.1182/blood.2020006520 32492712 PMC7378457

[6] Bikdeli B Madhavan MV Jimenez D Chuich T Dreyfus I Driggin E COVID-19 and Thrombotic or Thromboembolic Disease: Implications for Prevention, Antithrombotic Therapy, and Follow-Up. J Am Coll Cardiol (2020) 75(23):2950–73. 10.1016/j.jacc.2020.04.031 32311448 PMC7164881

[7] Tang N Li D Wang X Sun Z. Abnormal Coagulation Parameters Are Associated with Poor Prognosis in Patients with Novel Coronavirus Pneumonia. J Thromb Haemost (2020) 18(4):844–7. 10.1111/jth.14768 32073213 PMC7166509

[8] Panigada M Bottino N Tagliabue P Grasselli G Novembrino C Chantarangkul V Hypercoagulability of COVID‐19 Patients in Intensive Care Unit: A Report of Thromboelastography Findings and Other Parameters of Hemostasis. J Thromb Haemost (2020) 18(7):1738–42. 10.1111/jth.14850 32302438 PMC9906150

[9] Quintana-Díaz M Andrés-Esteban EM Ramírez-Cervantes KL Olivan-Blázquez B Juárez-Vela R Gea-Caballero V. Coagulation Parameters: An Efficient Measure for Predicting the Prognosis and Clinical Management of Patients with COVID-19. J Clin Med (2020) 9(11):3482. 10.3390/jcm9113482 PMC7692068 33126706

[10] Araya S Mamo MA Tsegay YG Atlaw A Aytenew A Hordofa A Blood Coagulation Parameter Abnormalities in Hospitalized Patients with Confirmed COVID-19 in Ethiopia. PLoS One (2021) 16(6):e0252939. 10.1371/journal.pone.0252939 34153056 PMC8216564

[11] Fogarty H Townsend L Ni Cheallaigh C Bergin C Martin‐Loeches I Browne P COVID19 Coagulopathy in Caucasian Patients. Br J Haematol (2020) 189(6):1044–9. 10.1111/bjh.16749 32330308 PMC7264579

[12] Ward SE Curley GF Lavin M Fogarty H Karampini E McEvoy NL Von Willebrand Factor Propeptide in Severe Coronavirus Disease 2019 (COVID‐19): Evidence of Acute and Sustained Endothelial Cell Activation. Br J Haematol (2021) 192(4):714–9. 10.1111/bjh.17273 33326604

[13] Helms J Tacquard C Tacquard C Severac F Leonard-Lorant I Ohana M High Risk of Thrombosis in Patients with Severe SARS-CoV-2 Infection: a Multicenter Prospective Cohort Study. Intensive Care Med (2020) 46(6):1089–98. 10.1007/s00134-020-06062-x 32367170 PMC7197634

[14] WHO. Clinical Management of COVID-19: Interim Guidance MAahwwipiic-M-O-. Geneva: WHO (2020).

[15] Ministry Of Health. Saudi MoH Protocol for Patients Suspected of/Confirmed with COVID-19 (2020). Available at: https://covidcalltohumanity.org/wp-content/uploads/2020/04/MOH-therapeutic-protocol-for-COVID-19.pdf (Accessed July 31, 2020).

[16] DeLong ER DeLong DM Clarke-Pearson DL. Comparing the Areas under Two or More Correlated Receiver Operating Characteristic Curves: a Nonparametric Approach. Biometrics (1988) 44(3):837–45. 10.2307/2531595 3203132

[17] Taylor FB Jr. Toh CH Hoots WK Wada H Levi M. Towards Definition, Clinical and Laboratory Criteria, and a Scoring System for Disseminated Intravascular Coagulation. Thromb Haemost (2001) 86(5):1327–30. 10.1055/s-0037-1616068 11816725

[18] Han H Yang L Liu R Liu F Wu K-l. Li J Prominent Changes in Blood Coagulation of Patients with SARS-CoV-2 Infection. Clin Chem Lab Med (2020) 58(7):1116–20. 10.1515/cclm-2020-0188 32172226

[19] Belen-Apak FB Sarıalioğlu F. Pulmonary Intravascular Coagulation in COVID-19: Possible Pathogenesis and Recommendations on Anticoagulant/thrombolytic Therapy. J Thromb Thrombolysis (2020) 50(2):278–80. 10.1007/s11239-020-02129-0 32372336 PMC7200048

[20] Guan W-j. Ni Z-y. Hu Y Liang W-h. Ou C-q. He J-x. Clinical Characteristics of Coronavirus Disease 2019 in China. N Engl J Med (2020) 382(18):1708–20. 10.1056/nejmoa2002032 32109013 PMC7092819

[21] Mao L Jin H Wang M Hu Y Chen S He Q Neurologic Manifestations of Hospitalized Patients with Coronavirus Disease 2019 in Wuhan, China. JAMA Neurol (2020) 77(6):683–90. 10.1001/jamaneurol.2020.1127 32275288 PMC7149362

[22] Lippi G Plebani M Henry BM. Thrombocytopenia Is Associated with Severe Coronavirus Disease 2019 (COVID-19) Infections: A Meta-Analysis. Clinica Chim Acta (2020) 506:145–8. 10.1016/j.cca.2020.03.022 PMC7102663 32178975

[23] Zhou F Yu T Du R Fan G Liu Y Liu Z Clinical Course and Risk Factors for Mortality of Adult Inpatients with COVID-19 in Wuhan, China: a Retrospective Cohort Study. The Lancet (2020) 395(10229):1054–62. 10.1016/s0140-6736(20)30566-3 PMC7270627 32171076

[24] Liu Y Yang Y Zhang C Huang F Wang F Yuan J Clinical and Biochemical Indexes from 2019-nCoV Infected Patients Linked to Viral Loads and Lung Injury. Sci China Life Sci (2020) 63(3):364–74. 10.1007/s11427-020-1643-8 32048163 PMC7088566

[25] Wardhani LFK Dewi IP Suwanto D Ambari AM Ardiana M. Case Report: Heparin-Induced Thrombocytopenia during COVID-19 Outbreak: the Importance of Scoring System in Differentiating with Sepsis-Induced Coagulopathy. F1000Res (2021) 10:469. 10.12688/f1000research.52425.2 34394916 PMC8356265

[26] Yin S Huang M Li D Tang N. Difference of Coagulation Features between Severe Pneumonia Induced by SARS-CoV2 and Non-SARS-CoV2. J Thromb Thrombolysis (2021) 51(4):1107–10. 10.1007/s11239-020-02105-8 32246317 PMC7124128

[27] Zou Y Guo H Zhang Y Zhang Z Liu Y Wang J Analysis of Coagulation Parameters in Patients with COVID-19 in Shanghai, China. Bst (2020) 14(4):285–9. 10.5582/bst.2020.03086 32350161

[28] Long H Nie L Xiang X Li H Zhang X Fu X D-dimer and Prothrombin Time Are the Significant Indicators of Severe COVID-19 and Poor Prognosis. Biomed Res Int (2020) 2020:6159720. 10.1155/2020/6159720 32596339 PMC7301188

[29] Connors JM Levy JH. COVID-19 and its Implications for Thrombosis and Anticoagulation. Blood (2020) 135(23):2033–40. 10.1182/blood.2020006000 32339221 PMC7273827

[30] Ranucci M Ballotta A Di Dedda U Baryshnikova E Dei Poli M Resta M The Procoagulant Pattern of Patients with COVID‐19 Acute Respiratory Distress Syndrome. J Thromb Haemost (2020) 18(7):1747–51. 10.1111/jth.14854 32302448 PMC9906332

[31] Lippi G Favaloro EJ. D-dimer Is Associated with Severity of Coronavirus Disease 2019: A Pooled Analysis. Thromb Haemost (2020) 120(5):876–8. 10.1055/s-0040-1709650 32246450 PMC7295300

[32] Chaudhary R Garg J Houghton DE Murad MH Kondur A Chaudhary R Thromboinflammatory Biomarkers in COVID-19: Systematic Review and Meta-Analysis of 17,052 Patients. Mayo Clinic Proc Innov Qual Outcomes (2021) 5(2):388–402. 10.1016/j.mayocpiqo.2021.01.009 PMC7869679 33585800

[33] Escher R Breakey N Lämmle B. ADAMTS13 activity, von Willebrand factor, factor VIII and D-dimers in COVID-19 inpatients. Thromb Res (2020) 192:174–5. 10.1016/j.thromres.2020.05.032 32505009 PMC7245313

[34] Escher R Breakey N Lämmle B. Severe COVID-19 Infection Associated with Endothelial Activation. Thromb Res (2020) 190:62. 10.1016/j.thromres.2020.04.014 32305740 PMC7156948

[35] Breakey N Escher R. D-dimer and Mortality in COVID-19: a Self-Fulfilling Prophecy or a Pathophysiological Clue? Swiss Med Wkly (2020) 150:w20293. 10.4414/smw.2020.20293 32459857

[36] Iba T Levy JH Connors JM Warkentin TE Thachil J Levi M. The Unique Characteristics of COVID-19 Coagulopathy. Crit Care (2020) 24(1):360. 10.1186/s13054-020-03077-0 32552865 PMC7301352

[37] Iba T Levy JH Raj A Warkentin TE. Advance in the Management of Sepsis-Induced Coagulopathy and Disseminated Intravascular Coagulation. J Clin Med (2019) 8(5). 10.3390/jcm8050728 PMC6572234 31121897

[38] Ladikou EE Sivaloganathan H Milne KM Arter WE Ramasamy R Saad R Von Willebrand Factor (vWF): Marker of Endothelial Damage and Thrombotic Risk in COVID-19? Clin Med (2020) 20(5):e178–e182. 10.7861/clinmed.2020-0346 PMC7539718 32694169

[39] Al Otair H AlSaleh K AlQahtany FS Al Ayed K Al Ammar H Al Mefgai N The Level of vWF Antigen and Coagulation Markers in Hospitalized Patients with Covid-19. Jbm (2021) Vol. 12:809–17. 10.2147/jbm.s318940 PMC8416187 34512061

[40] Martín-Rojas RM Pérez-Rus G Delgado-Pinos VE Domingo-González A Regalado-Artamendi I Alba-Urdiales N COVID-19 Coagulopathy: An In-Depth Analysis of the Coagulation System. Eur J Haematol (2020) 105(6):741–50. 10.1111/ejh.13501 32749010 PMC7436538

[41] Lelas A Greinix HT Wolff D Eissner G Pavletic SZ Pulanic D. Von Willebrand Factor, Factor VIII, and Other Acute Phase Reactants as Biomarkers of Inflammation and Endothelial Dysfunction in Chronic Graft-Versus-Host Disease. Front Immunol (2021) 12:676756. 10.3389/fimmu.2021.676756 33995421 PMC8119744

[42] Tichelaar V Mulder A Kluin-Nelemans H Meijer K. The Acute Phase Reaction Explains Only a Part of Initially Elevated Factor VIII:C Levels: a Prospective Cohort Study in Patients with Venous Thrombosis. Thromb Res (2012) 129(2):183–6. 10.1016/j.thromres.2011.09.024 21992898

[43] Gragnano F Sperlongano S Golia E Natale F Bianchi R Crisci M The Role of von Willebrand Factor in Vascular Inflammation: From Pathogenesis to Targeted Therapy. Mediators Inflamm (2017) 2017:5620314. 10.1155/2017/5620314 28634421 PMC5467347

